# Online ethnography of breastfeeding discussions in social networking sites for Hong Kong mothers

**DOI:** 10.1002/fsn3.3796

**Published:** 2023-11-20

**Authors:** Toma Chun Yip Wong, Karene Hoi Ting Yeung, Venera R. Khalikova, Lai Ling Hui, Ka Ming Chow, Esther Yuet Ying Lau, E. Anthony S. Nelson

**Affiliations:** ^1^ Department of Paediatrics, Faculty of Medicine The Chinese University of Hong Kong New Territories Hong Kong; ^2^ Department of Anthropology The Chinese University of Hong Kong New Territories Hong Kong; ^3^ Department of Food Science and Nutrition The Hong Kong Polytechnic University Kowloon Hong Kong; ^4^ The Nethersole School of Nursing, Faculty of Medicine The Chinese University of Hong Kong New Territories Hong Kong; ^5^ Department of Psychology The Education University of Hong Kong New Territories Hong Kong; ^6^ School of Medicine The Chinese University of Hong Kong Shenzhen Guangdong China

**Keywords:** breastfeeding, Hong Kong, online ethnography, social networking sites

## Abstract

With the growth of social networking, parents are increasingly sharing their experiences and opinions or seeking help with childcare through online platforms. This study explored breastfeeding‐related topics that Hong Kong mothers raise on social networking sites and how other mothers respond; and how these sites could be a facilitator or barrier to breastfeeding. An online ethnographic approach was used to collect breastfeeding‐related discussions (posts and responses) among mothers from three sources: two closed moderated Facebook groups with more than 1000 members, and one open unmoderated forum (Baby Kingdom) (26 December 2021–26 May 2022). Posts not related to breastfeeding (e.g., about formula feeding only) were excluded. Data were collected by a nonparticipatory approach to avoid disrupting the dynamics of the groups. In total, 131 original posts and their 802 responses were collected, of which the common topics discussed were breastfeeding technique, breastfeeding‐related health issues, breastfeeding mothers returning to work, and COVID‐19 vaccination/infection during breastfeeding. The responses to the queries on breastfeeding technique and health issues in the closed groups were mostly about sharing breastfeeding knowledge and health information to provide timely emotional support and practical solutions. Although similar responses were observed in the open forum, sharing experiences in using formula milk were frequently observed in the responses to posts related to breastfeeding. Social networking sites could be facilitators and barriers to breastfeeding. The potential for infant formula promotion in open forums requires further monitoring and evaluation. Moderation and support from trained professionals or peers could be important.

## Graphical abstract

This online ethnographic study explored breastfeeding discussions among Hong Kong mothers on social networking sites. Common topics discussed on two closed and one open social networking sites were breastfeeding technique issues, breastfeeding‐related health issues, and COVID‐19 vaccination/infection concerns. Closed Facebook groups moderated by mothers with breastfeeding experience can facilitate breastfeeding by sharing breastfeeding knowledge and techniques and providing emotional support. Open unmoderated social networking sites have the potential for infant formula promotion that requires further evaluation.

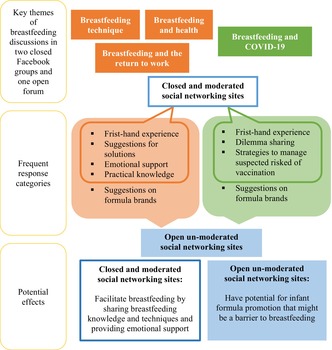

## 1 BACKGROUND

Breastfeeding benefits both mother and baby (Centers for Disease Control and Prevention, 2023), for example, by reducing the risk of infection in infants and premenopausal breast cancer in mothers (Stuebe, 2009). The World Health Organization (WHO) recommends mothers initiate breastfeeding within the first hour of their infant's lives, exclusively breastfeed for at least 6 months, and continue breastfeeding for up to 2 years and beyond (World Health Organization, n.d.). The Hong Kong Special Administrative Region (HKSAR) Government emphasizes the advantages of breastfeeding and commits to providing professional and social support to breastfeeding mothers (Department of Health, n.d.).

A local 2020 survey reported that 87% of postnatal women initiated breastfeeding before hospital discharge (Department of Health, n.d.). However, only 24% of mothers continued breastfeeding at 12 months and the exclusive breastfeeding rate dropped from 29% at 1 month to 22% at 6 months. Local studies show that Hong Kong women's decision to breastfeed is influenced by complex social, cultural, and practical factors (Tarrant et al., 2002). In particular, lack of breastmilk and returning to work were the most common reasons given for discontinuing breastfeeding (Shakya et al., 2017), whereas positive correlations have been noted between peer support and breastfeeding duration (Gill et al., 2007; Shakya et al., 2017).

Online communication through social networking sites has become one of the more popular ways for parents to share experiences and opinions or seek help with childcare regardless of geographical location (DeHoff et al., 2016; Frey et al., 2022). Parents posted and responded to breastfeeding questions in online breastfeeding support groups in Australia and the United Kingdom (Bridges, 2016; Bridges et al., 2018; Wagg et al., 2019). A wide range of topics were discussed in these groups which provided informational and emotional support to parents. To the best of our knowledge, there has been no similar study in Hong Kong. We set out to explore breastfeeding‐related topics that mothers raise on closed and open social networking sites and how other mothers respond to those discussions to understand whether these interactions could be a facilitator or barrier to breastfeeding.

## 2 METHODS

### 2.1 Study design

The study used an online ethnographic approach to collect breastfeeding‐related discussions among mothers by observing their interactions on social networking sites. Ethnography is a research method of studying people's behavior in a naturally occurring and ongoing setting. It emphasizes the immersion of researchers into the field of study, to watch and listen to the insider perspectives and provide interpretive explanations of the interactions within a community or cultural group (Watson‐Gegeo, 1988). In anthropology, the two primary methods of ethnography are participant observation and in‐depth interviews, and the same methods are ideally used online. However, ethnographic methods have been used across disciplines, with numerous methodological adaptations that include nonparticipatory approaches and unobtrusive observation (Garcia et al., 2009). This study followed a nonparticipatory approach to avoid disrupting the dynamics of the studied groups. The research staff did not participate in any of the discussions during the data collection period.

### 2.2 Data collection

Breastfeeding‐related discussions (posts and responses) from two closed Facebook groups and one open forum from 26 December 2021 to 26 May 2022 were collected. Posts not related to breastfeeding (e.g., about formula feeding only) were excluded. All discussions were in written Cantonese and were translated into English by researchers for reporting.

There are four active peer‐led Facebook groups in Hong Kong for breastfeeding discussions in written Cantonese with more than 1000 members. All are closed groups where only approved people can join. Members can only post questions and discuss topics related to breastfeeding. There are rules and guidelines for discussion to ensure a friendly environment for mutual support among mothers and the quality of the discussion. Meanwhile, there are no specific rules in the open forum regarding breastfeeding discussion. People can discuss anything freely as long as it is done in respectful manner.

We approached the administrators of the four closed Facebook groups and two consented to participate in the study. One is organized voluntarily by mothers who have breastfeeding experience and the other by a nonprofit organization. Both groups are moderated by mothers with breastfeeding experience and have certified lactation consultants participating and answering questions from time to time.

Besides the two Facebook groups, breastfeeding‐related discussions in one of Hong Kong's most popular open forums, “Baby Kingdom”, were also collected. This open forum is mainly used by parents to discuss parenting topics. Chinese words that are linked to breastmilk and breastfeeding (人奶/母乳/奶) were used to search for related posts.

### 2.3 Data analysis

Thematic analysis was used to have a structural and thorough understanding of the breastfeeding topics discussed on the social networking sites by grouping posts into different themes and of the reactions of other mothers to the posts by coding the responses. Each post can be classified into more than one theme based on the content raised by the mothers. The coding was done by one researcher and another researcher reviewed the codes. Discussions were conducted among the two researchers for consensus on discrepancies found.

### 2.4 Ethics approval

This study was approved by the Survey and Behavioral Research Ethics Committee—The Chinese Univeristy of Hong Kong (Reference No. SBRE‐21‐0576). Consent was obtained from the administrators of the two Facebook groups for data collection. We provided an option to put up a pinned post in the group to describe the study details so that the group members could be aware of the research during the period, and one group posted this.

## 3 RESULTS

In total, 131 original posts and 802 responses to these posts were collected from the three social networking sites. The majority of the posts (94 posts, 72%) were from the open forum *(indicated by “A” in the quotations below)*, and 17 and 20 posts were from the two closed Facebook groups *(indicated by “B” and “C” in the quotations below)*. These included all posts from the two closed groups within the timeframe as all their posts were breastfeeding related; and only breastfeeding‐related posts from the open forum were collected. Four main themes of breastfeeding topics were identified from 122 posts and included in the analysis. They were (i) breastfeeding technique; (ii) breastfeeding and health; (iii) breastfeeding and the return to work; and (iv) breastfeeding and COVID‐19. Detailed explanations of each theme are described in Table 1.

**TABLE 1:** Definition of the breastfeeding themes of the study.

Theme	Description
Breastfeeding technique	Issues related to technique of breastfeeding, e.g., strategies of breastfeeding, frequency of feeds, use of breast pumps, appeasing babies, relactation, low supply of breastmilk, weaning, diet, and medical supplements
Breastfeeding and health	Questions concerning mothers' health such as breastfeeding‐related pain, mastitis, blocked ducts, swelling, stress, menstruation, and pregnancy Questions concerning breastfed babies' health such as weight gain/growth, body temperature, digestive issues, and neonatal jaundice
Breastfeeding and the return to work	Discussions related to breastfeeding mothers returning to work, e.g., emotional issues and weaning due to returning to work
Breastfeeding and COVID‐19	Discussions related to COVID‐19 vaccination and infection during breastfeeding

### 3.1 Breastfeeding technique

Breastfeeding technique was the most common topic on social networking sites (58%). It included discussions about strategies for expressing breastmilk, frequency of breastfeeding, solutions for a low supply of breastmilk, weaning, etc.

Mothers who asked these questions were looking for advice from experienced mothers when they struggled with handling breastfeeding. New mothers encountered different breastfeeding problems and could not identify the associated causes easily, so the participation of experienced mothers in the discussions helped unravel their problems. For example:

I felt pain in my nipples when pumping, and the amount of breastmilk dropped compared to a month ago. (B35)

The experienced mothers provided insights on potential causes, solutions, and recommendations for the problems based on their experience. They asked problem‐solving‐oriented and straightforward questions to obtain precise information for providing suitable recommendations. In the above example, several mothers asked for more details such as “Which brand of pump are you using? Which pumping mode did you select? How long did you pump each time?” In the end, the mother realized that the pumping mode of the breast pump she had set might be too strong and she decided to follow the advice from other mothers in using the breast pump.

Even though new mothers are usually help seekers, some of them provided details of the problems to facilitate the discussion. In a post (B68), a mother consulted for the cause of her baby's irritable behavior. After returning to work, she changed from breastfeeding her baby directly exclusively to a mix of bottle feeding and direct breastfeeding. When she breastfed the baby directly at night, the baby became irritable, bit her nipples, and even vomited. She provided background information for efficient responses by other mothers, for example, the amount of breastmilk she had and her feeding position. She also suspected that her baby was experiencing nipple confusion.

A major difference between the closed and open‐access sites in terms of the types of responses when mothers asked questions about breastfeeding technique was observed. In the closed groups, the most frequent type of response was sharing of breastfeeding knowledge. For instance, a mother of a 2‐month‐old baby felt that the amount of breastmilk dropped compared to a month before and questioned:

My baby is already one month old now. I cannot increase my breastmilk supply, can I? Is the amount of breastmilk already fixed? (B25)

Experienced mothers responded that breastmilk supply could still be boosted in the first couple of months and provided practical tips on how to increase the supply of breastmilk. In contrast, sharing personal experiences of using formula milk was the most common response when mothers asked about weaning in the open forum. Some respondents even suggested specific brands of formula milk:

Don't be too stressed even if you do not breastfeed. Your baby can still grow healthily with formula milk. I gave formula milk of [brand] to my baby and my baby did not get sick, has a good digestive system and can play and sleep well. I am already very satisfied. (A23)

### 3.2 Breastfeeding and health

Health‐related concerns of both breastfeeding mothers and breastfed babies were a common topic raised in the studied groups (19%). Health problems of mothers included physical and mental health such as breastfeeding caused pain, mastitis, blocked ducts, breast swelling, stress, etc. For their babies, breastfeeding mothers were mainly concerned about their weight gain/growth and digestive system. Mothers showed their worries and psychological stress when talking about health problems.

The health problems raised by mothers on social networking sites also happened in other experienced mothers. Below is an example from the open forum titled: “Breastfeeding is frustrating”. A breastfeeding mother was experiencing blocked ducts and mastitis. She felt discouraged about her breastfeeding experience because of the pain she endured.

My baby is now 2.5 months old. I have been feeding my baby with expressed breastmilk. This month, I tried to breastfeed my baby directly twice a day and bottle‐feed my baby with defrosted breastmilk four times a day. What discouraged me is that I always have mastitis and blocked ducts. I also found some white spots on my nipples these two days. I don't know if it's a problem with the breast pump or a posture problem of breastfeeding but I've read a lot of information on the internet and I think my baby drinks correctly. My nipples are very painful all the time. I want to know if I should accept it's my fate. Is blocked duct unavoidable in some people like me? I will have to return to work next month and I really want to continue breastfeeding for 6 months. However, if mastitis and blocked duct happen every two weeks, I would give up… (A46)

The responses were a mix of suggestions and emotional support. The respondents encouraged the mother to try her best to breastfeed by showing empathy, and also suggested formula feeding. They were concerned about the psychological health of the mother and thought stopping breastfeeding could be the last resort for this mother.

I feed my baby only with expressed breastmilk and I take lecithin every day. So far no blocked duct. Would you consider trying it? (A46)

First of all, don't be too stressed. I had mastitis before too so I know how painful it is. […] It was caused by not pumping for too long and I relied on lecithin and sucking by the baby. […] Continuing breastfeeding is hard. Don't be so stressed. [Your baby] would also be healthy if you decide to give formula milk. Do your best [in exclusive breastfeeding] and stay positive is the most important. (A46)

Although breastfeeding is the best option, your psychological health is also important. After your baby was born, you have a lot to manage, especially if you are a new mother. Not stressing yourself is the most important. You can give formula milk if no breastmilk. I stopped breastfeeding when I was back to work but in return, I can have time to spend with my baby after work. My baby and I are happy with that. (A46)

Some mothers posted questions on their babies' health and checked if this was common to other breastfed babies:

Hello everyone, I am a new mother. My baby is now 2.5 weeks old. I have exclusively breastfed my baby since birth. He has approximately 8 meals and more than 6 wet diapers per day. However, his weight is still lower than his birth weight. Did anyone have the same experience? I am now very hesitant to continue breastfeeding my baby. Thanks. (C05)

Several respondents indicated that they had similar experiences and provided concrete suggestions to the mother to evaluate the problem such as confirming that the baby actually drank the breastmilk by feeling the breasts softened after feeding and checking the volume of breastmilk by pumping. One respondent quoted scientific knowledge of how epidural and oxytocin will affect babies' bodyweight if they were used during delivery.

Do you have gestational diabetes? According to Jack Newman's book, if epidural or oxytocin was used during delivery, some chemicals would be passed to the baby. This would affect the measured birth weight, which is not its real weight. […] (C05)

The mother who created the post replied:

Yes, I had gestational diabetes. The same accelerant was used at delivery. Is it, as you said, because of the above two points [having gestational diabetes and the use of epidural and oxytocin during delivery], the measured birth weight would be higher than a ‘real’ one? […] However, the doctor at the health centre did not consider gestational diabetes as a potential cause of it. (C05)

The responses to the health issues of breastfeeding in the closed groups were commonly sharing of health information and breastfeeding knowledge. Sharing personal experiences supporting breastfeeding was also observed in those groups. However, in the open forum, sharing personal experiences in using formula milk was frequently observed.

### 3.3 Breastfeeding and the return to work

Returning to work was a less common topic on the social networking sites and only six posts were found on the open forum. Mothers asked for advice on expressing breastmilk or formula feeding. Some mothers also asked for ideas on brands of formula milk that are suitable for breastfed babies.

### 3.4 Breastfeeding and COVID‐19

The fifth wave of the COVID‐19 pandemic in Hong Kong fell within the data collection period. The HKSAR Government implemented the “Vaccine Pass” on 21 February 2022 (the middle of the data collection period), that is, COVID‐19 vaccination was required to enter certain venues (The Government of Hong Kong Special Administrative Region, n.d.). Some companies also required their staff to be vaccinated for work. Some pregnant women and breastfeeding mothers were concerned about the side effects of the vaccines on their babies. According to our data, 23% of posts were related to COVID‐19 vaccination and infection during breastfeeding. Mothers sought suggestions on receiving COVID‐19 vaccines during breastfeeding and managing breastfeeding after vaccination and infection.

Among the three sites, mothers generally initiated a COVID‐19‐related discussion by asking a yes/no question, which showed they were eager to get a quick answer:

Subject of post: Should breastfeeding mothers receive [COVID‐19] vaccines?

Should I get the vaccine? My baby is now only a few months old. Would there be any side effects to my baby? (A01)

This mother further explained the reason for raising this question when replying to another mother facing the same dilemma. They were worried about being infected and understood that vaccination is one possible intervention, but they were also concerned about the side effects of the vaccine. They wanted to consult others' experiences and opinions before making a decision.

The severity of the fifth wave [of COVID‐19] concerns me. I am not sure about its [the vaccine's] impacts on my baby and whether I can tolerate its reactions on myself. Therefore, I am struggling… (A01)

The most common responses to this kind of question were sharing positive experiences of vaccination and sharing the same dilemma:

I received two doses of the vaccine in December and January. I am still breastfeeding my baby and feeding one meal of formula milk at night. My baby is now 11 weeks old and I didn't see any side effects [on the baby after vaccination]. My work nature is potentially at high risk of COVID‐19 infection. I was forced to be vaccinated. (A10)

First, I am worried about having severe reactions by the vaccine. Second, I am more or less worried about whether the vaccine will affect the breastmilk. Although current data shows that it is fine to receive the vaccine, no one knows about what will happen in the future. Third, I am worried there will be another peak of infection in the New Year, so I decided to receive the vaccine and stop breastfeeding. (A60)

I am sad whenever I think of stopping breastfeeding because of vaccination. I needed to do so for work. There is no other way out. If I don't have to work, I would avoid visiting places that require vaccination and insist on breastfeeding as long as I can. (A60)

When facing the dilemma, some mothers received the vaccines and continued breastfeeding, some decided to stop breastfeeding for vaccination, and some stopped breastfeeding for a few days after vaccination.

Foreign literature mentioned the antibodies can be transmitted to babies [through vaccination] and there is no side effect to them. I recently received my first dose [of COVID‐19 vaccine] and stopped breastfeeding for 4 days. I started breastfeeding three days ago and nothing abnormal [happened to my baby]. (A01)

### 3.5 Formula milk‐related discussion

**FIGURE 1:**
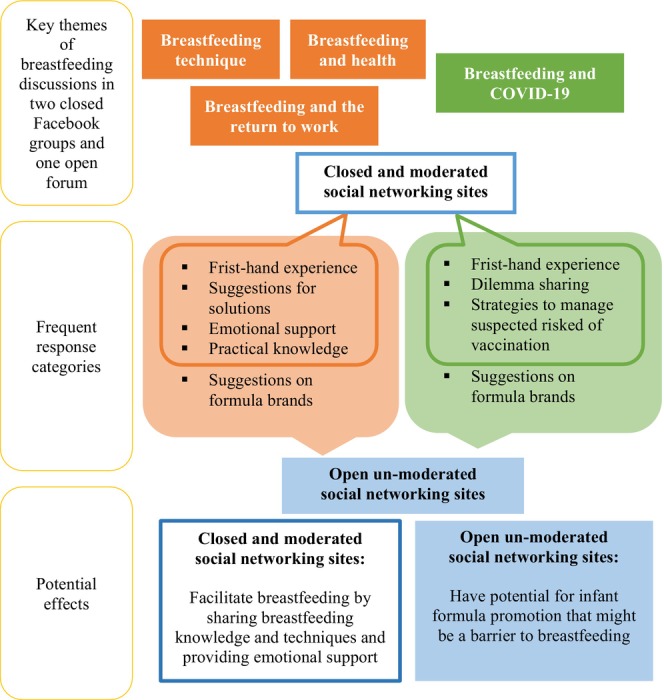
Key themes of breastfeeding discussions, frequent responses, and potential effects identified in the study.

Formula‐related discussions were observed in some breastfeeding‐related posts and all were from the open forum (Figure 1). In total, there were 31 posts related to formula feeding, of which 12 posts were asking for suggestions on formula brands by breastfeeding mothers and 17 discussions included formula brand recommendations without anyone asking for it. Similar responses were also observed on formula brand recommendation. The respondents usually shared their personal experiences with the use of a particular brand of infant formula.

My baby tried [band name], and I experienced a smooth transition from breastmilk to formula milk. I tried the formula milk myself and I think the taste and scent were good.

## 4 DISCUSSION

During the data collection period, more than 90% of the breastfeeding‐related topics discussed in the studied groups were breastfeeding technique, breastfeeding‐related health issues, and COVID‐19 vaccination/infection during breastfeeding. Mothers posted queries in the groups for seeking advice from other mothers who might have similar experiences. For all topics, respondents to the posts usually shared their own experiences as suggested solutions to the queries. In addition, for the topics of breastfeeding technique and health issues, respondents tried to help answer the queries by sharing knowledge and information. Emotional support was in particular common in health‐related queries. For the posts related to COVID‐19, respondents typically shared the same dilemma, even if they did not provide a specific answer.

Based on the topics raised by mothers on social networking sites, we can identify some potential barriers or obstacles to breastfeeding for Hong Kong mothers. As noted in the literature, insufficient breastmilk and returning to work are the most common factors reported by mothers that lead to supplementation of formula milk (Garcia et al., 2009; Tarrant et al., 2002). In this ethnographic study, a low supply of breastmilk was frequently raised in the studied groups which confirmed the perception of insufficient breastmilk as a major barrier to breastfeeding. Although there were not many posts about returning to work, it is believed that this is still a barrier to breastfeeding. Among five of six discussions on the return to work, mothers asked for suggestions of brands of infant formula instead of asking for advice on sustaining breastfeeding after returning to work. Tarrant et al. (2010) also argued that Hong Kong mothers did not want to burden others at their work because of their need to express breastmilk. This may be one of the reasons why there were few concerns raised on social networking sites. In addition, mastitis and blocked ducts were also observed as obstacles to breastfeeding since the pain that mothers suffered discouraged them from continuing breastfeeding. Since the data collection period fell within the COVID‐19 pandemic, COVID‐19 vaccination and related policies were barriers to breastfeeding. It appeared that some mothers opted for stopping breastfeeding their babies completely or temporarily after vaccination.

Social networking sites can be a facilitator of breastfeeding. Mothers benefitted from breastfeeding knowledge and practical information shared by other mothers on these sites. Especially in the closed groups, trained peer breastfeeding supporters were available to respond to the mothers having queries or problems. Some respondents referred to evidence such as information from authorities and academic literature when responding to the posts. Also, the social networking sites provide an opportunity for mothers encountering different challenges during their breastfeeding journey to express their concerns and obtain timely emotional support.

However, it is difficult to control the reliability of the information shared and ensure a breastfeeding‐friendly environment on open‐access sites. In such cases, these sites, without trained peer breastfeeding supporters, might be a barrier to breastfeeding. The discussions of formula feeding related to breastfeeding‐related posts were only observed in the open forum since the closed groups discourage this to ensure a breastfeeding‐friendly environment in the groups. It was also possible that mothers who were less committed to breastfeeding did not join the breastfeeding groups. With no restriction to access in the open forum, there could be immersion of representatives from formula companies or social influencers who receive benefits for promoting particular products in the online discussion. It is impossible to know to what extent questions asking for suggestions about infant formula brands are planted to obtain responses that recommend specific brands. Although the identities of the respondents cannot be confirmed, similar responses to the topics of breastfeeding technique and health issues were observed. They shared their positive experience in using a specific brand of infant formula and claimed that the specific formula brand was good for transitioning from breastfeeding to formula feeding.

There are some limitations in this study. First, two other breastfeeding groups that are closed to the public with more than 1000 members did not agree to join the study. We cannot be sure that all types of breastfeeding topics and responses were captured in this study. Second, there are WhatsApp groups among mothers to discuss childcare‐related issues that were not possible for the researcher to join. Third, it is possible that the mothers who are less committed to breastfeeding did not ask questions on social networking sites or were not interested in joining the breastfeeding groups in the first place. Therefore, the questions and concerns of those mothers might not have been captured in this study. Fourth, with the proliferation of other social networking apps such as Instagram and Snapchat among the young people, Hong Kong mothers aged in their mid‐20s might be underrepresented since Facebook is not their only platform for socializing. Fifth, since this study was designed to be based on nonparticipatory observation and did not include interviews, the demographic details of mothers active in these sites are unknown. Therefore, the representativeness of this sample cannot be confirmed, and the broader sociocultural context in which these mothers live cannot be assessed. Sixth, online ethnography does not provide a full picture of people's lives, and many researchers have emphasized the need to supplement the data collected online with offline research. A further ethnographic study with face‐to‐face interviews and participation is thus required to better understand the life experiences, concerns, and challenges of breastfeeding mothers in Hong Kong.

This study has shown that it is common for mothers to seek help on breastfeeding problems on social networking sites and these sites can also serve as platforms for mothers to express their stress and negative emotions related to breastfeeding. The barriers to breastfeeding identified from the discussion in these sites should be incorporated into the routine health education and promotion activities related to breastfeeding. It is difficult or impossible to control the discussion in the open‐access platforms but the government can financially support more breastfeeding groups so that qualified and trained peer breastfeeding supporters can be arranged to provide timely and suitable support to mothers. There has already been face‐to‐face support at birthing hospitals and hotlines of breastfeeding support. Since new generations of mothers rely more on online platforms, online support is important to mothers in managing to breastfeed and solving some health‐related problems. The government should consider implementing online channels for mothers to seek help, for example, one‐to‐one conversations through applications such as WhatsApp, WeChat, and Telegram.

To conclude, the common topics being discussed in the three social networking sites in the study were breastfeeding technique, breastfeeding‐related health issues, and COVID‐19 vaccination/infection during breastfeeding. Breastfeeding mothers can acquire breastfeeding knowledge, practical information, and emotional support from peers on social networking sites. These sites could be facilitators and barriers to breastfeeding. The potential for infant formula promotion in open forums requires further monitoring and evaluation. Moderation and support from trained professionals or peers could make a difference. Pregnant women and mothers should be alerted to the difference between open and closed sites.

## AUTHOR CONTRIBUTIONS

**Toma Chun Yip Wong:** Conceptualization (equal); data curation (lead); formal analysis (equal); investigation (equal); methodology (equal); writing – original draft (lead); writing – review and editing (equal). **Karene Hoi Ting Yeung:** Conceptualization (equal); formal analysis (equal); investigation (equal); methodology (equal); project administration (lead); supervision (equal); writing – original draft (supporting); writing – review and editing (equal). **Venera R. Khalikova:** Conceptualization (equal); formal analysis (supporting); methodology (equal); writing – review and editing (equal). **Lai Ling Hui:** Conceptualization (equal); formal analysis (supporting); methodology (equal); writing – review and editing (equal). **Ka Ming Chow:** Formal analysis (supporting); writing – review and editing (equal). **Esther Yuet Ying Lau:** Formal analysis (supporting); writing – review and editing (equal). **E. Anthony S. Nelson:** Conceptualization (equal); formal analysis (supporting); funding acquisition (lead); methodology (equal); supervision (equal); writing – review and editing (equal).

## CONFLICT OF INTEREST STATEMENT

The authors declare that they have no conflicts of interests.

## ETHICS STATEMENT

This study was approved by the Survey and Behavioral Research Ethics Committee—The Chinese University of Hong Kong (Reference No. SBRE‐21‐0576).

## Data Availability

Data from the open forum are accessible to researchers upon request for data sharing to the corresponding author. Request for data from the closed Facebook groups requires approval by the administrators of the respective groups.
